# A limited overlap of interactions between the bacterial community of water and sediment in wetland ecosystem of the Yellow River floodplain

**DOI:** 10.3389/fmicb.2023.1193940

**Published:** 2023-06-22

**Authors:** Zhiguang Han, Cong Wang, Binghai Lei, Nan Hui, Yanyan Yu, Yu Shi, Junqiang Zheng

**Affiliations:** ^1^Yellow River Floodplain Ecosystems Research Station, School of Life Sciences, Henan University, Kaifeng, Henan, China; ^2^Department of Civil Engineering and Architecture, Henan University, Kaifeng, Henan, China; ^3^International Joint Research Laboratory for Global Change Ecology, School of Life Sciences, Henan University, Kaifeng, Henan, China; ^4^School of Agriculture and Biology, Shanghai Jiao Tong University, Shanghai, China

**Keywords:** wetland ecosystem, Yellow River floodplain, water and sediment, microbial communication, bacterial community

## Abstract

**Introduction:**

Aquatic ecosystems in floodplains provide homes for a variety of active bacterial populations. However, the coexistence pattern of bacterial communities of water and sediment in these ecosystems is unclear.

**Methods:**

In the present study, Illumina Mi-Seq sequencing were to assess bacteria's co-occurrence patterns in the water and sediment of different time dynamics and plant communities of the Yellow River floodplain ecosystem.

**Results and discussion:**

The results showed that compared to water, the α-diversity of the bacterial community was way greater in sediment. The bacterial community structure significantly differed between water and sediment, and there was a limited overlap of interactions between the bacterial community of water and sediment. In addition, bacteria in water and sediment coexisting show different temporal shifts and community assembly patterns. The water was selected for specific groups of microorganisms that assemble over time in a non-reproducible and non-random way, whereas the sediment environment was relatively stable, and the bacterial communities were gathered randomly. The depth and plant cover significantly influenced the structure of a bacterial community in the sediment. The bacterial community in sediment formed a more robust network than those in water to cope with external changes. These findings improved our comprehension of the ecological trends of water and sediment bacterium colonies coexisting enhanced the biological barrier function, and the capacity of floodplain ecosystems to provide services and offered support for doing so.

## 1. Introduction

The floodplain is a region of transition that connects riverside habitats. It located in the extension area of water-land interaction (Allison et al., 1998). The floodplain ecosystem is important to the biogeochemical cycle coupling between land and river. In this unique environment, the water is the carrier of biological and abiotic substances, and the sediment is the sink or source of the nutrient cycle. Microbial populations in sediment and water are crucial to systems such as the biogeochemical cycle, biotransformation of contaminants, and greenhouse gas emissions (Fillinger et al., 2019). The common consensus is that the microbial community in sediment is developed through long-term accumulation, sedimentation, and erosion (Du et al., 2020). Therefore, fluctuation, structural changes, and an abundance of sediment changes in microbial communities parallel biological and soil factors (such as plant community composition and soil attributes). The microbial community in water comes from rainfall, groundwater, and sediment. Due to regional variations in hydrological circumstances caused by diverse terrain, water quality, land use, and geomorphology, microbial communities in water vary greatly between different locations (Altermatt, 2013; Mari et al., 2014). Based on this, microbial communities' diversity, composition, and environmental elements in sediment and water may be significantly different (Xia et al., 2014; Cloutier et al., 2015; Nevers et al., 2020). The composition and diversity of microbial communities provide a reliable reference value for the health and stability of the ecology in the floodplain. Correspondingly, one of the hotspots in wetland ecosystem research has always been the composition and structure change pattern of bacteriological species in sediment and water.

Studies show that co-existing bacterial communities in sediment and water exhibit various and diverse environmental vulnerabilities and preferences (Zhang et al., 2021). The diversity, composition, and structure of bacterial communities can be affected by abiotic (including biological matter, hydrological conditions, and temperature) and biological factors (such as typical plants) (Zhang et al., 2014; Li et al., 2019; Xia et al., 2020). Due to the difference of habitats, there are significant differences in the diversity and composition of plankton bacterial communities and sediment bacterial communities in lakes (Jiao et al., 2020; Zhang et al., 2021). In addition, plant species are selective about the microorganisms in the rhizosphere and planktonic bacteria, and the existence of plants can alter the aquatic environment's horizontal and vertical heterogeneity to influence microbial ecosystem communities (Fan et al., 2016; Wang et al., 2018). Our previous research shows that in drought periods, the soil bacterial community structure under different plant covers has significant differences. In flood periods, however, it is unclear how the bacterial community performance and dynamic response model of coexisting water and sediment bacteria under different plant covers differ (Yu et al., 2020). Moreover, the floodplain has obvious erosion and deposition, which leads to soil stratification. Soil stratification also creates significant differences in soil physical and chemical properties at different depths (Yu et al., 2020). Whether the physical and chemical properties of sediment also have significant differences with depth, and whether this difference will affect microbial communities, is unclear. Understanding the above issues will enable us to comprehend the floodplain's role as a barrier and ecological service.

The Yellow River floodplain, an important ecosystem, is profoundly impacted by aquatic and coastal ecosystem services and sustainable socio-economic development related to human activity and climate change (Costanza et al., 2014). Limited by the reservoir construction and the diversion control projects on both sides of the river channel, there is a special type of wandering floodplain in the Yellow River's reduced and intermediate reaches, especially in the height of the flood season, which is affected by the river channel (Zhang et al., 2016). Therefore, the water and sediment conditions of this kind of wandering floodplain are more uncertain, and the hydraulic driving mechanism of microorganisms re more complex. Here, we investigated the dynamic change of bacterial diversity and community structure in different plants and explored the co-occurrence patterns of bacterial communities in the water and sediment of the Yellow River floodplain ecosystem. In this research, we postulated that (1) The diversity and community structure of bacteria in sediment and water differ greatly from one another and are driven by different environmental factors and (2) Soil depth and plant type will profoundly affect the bacterial community structure of sediment.

## 2. Methods and materials

### 2.1. Study design and collection of sample

**Figure 1:**
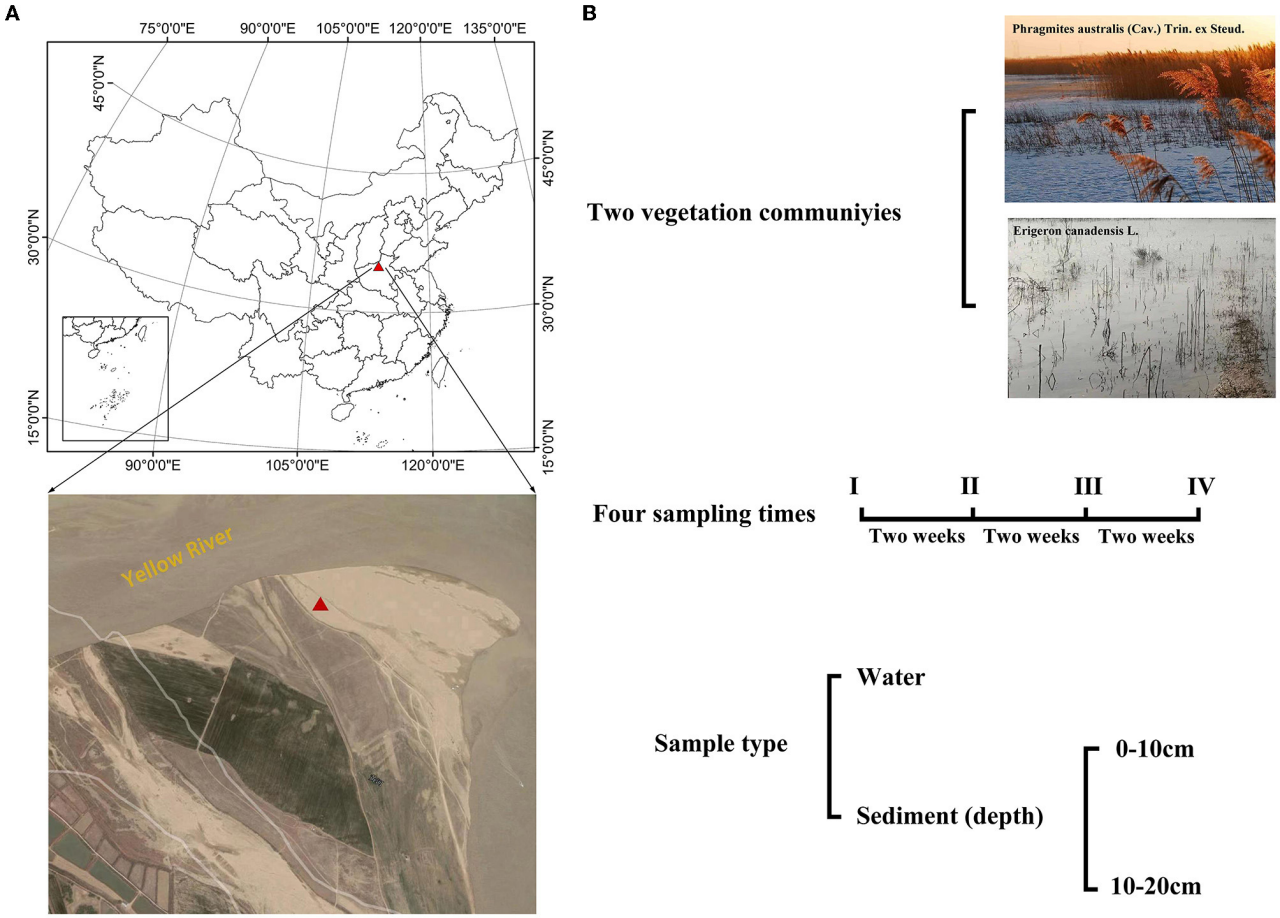
**(A)** Maps of the sampling sites in this study. **(B)** Water and sediment sampling methods.

The study was done in Henan, China, at the Yellow River Floodplain Ecosystems Research Station, located at 34°59'65” N and 113°25'05” E. Two plant communities (*Phragmites australis* (Cav.) Trin. ex Steud. and *Erigeron canadensis* L.) were selected from the floodplain. These two plant communities are the dominant species in the Yellow River floodplain ecosystem system. Three plots were set up in each plant community (Figure 1). In August and September 2020, water and sediment samples were collected every 2 weeks, four times in total. Sediment drilling was performed twice at 0–10 cm for the surface and 10–20 cm for the subsurface, and three points from each plot were selected and combined. In total, 48 sediments were taken (2 plant communities × 3 plots × 2 depths × 4 times). At each sampling plot, a 1.5 L water sample was collected, and the sum of 24 water samples was taken (2 plant communities × 3 plots × 4 times).

The samples were delivered to the lab in sanitized polythene bags and on ice. Some water portions were passed through a 0.45 μm filter for physical and chemical property determination. Another portion of the water proceeded through a 0.22 μm filter (Merck Millcore Ltd.) and was employed in order to identify the bacterial structural community. Two groups of sediment were distinguished. To ascertain the composition of the bacterial community, one half was kept at a controlled temperature of −80 °C. For the physicochemical inspection, the residual sample was air dried at an ambient temperature.

### 2.2. Physical-chemical evaluation

The total nitrogen and carbon contents of the sediment were examined using the Vario Max CNS elemental analyzer (Elementar Analysensysteme GmbH, Hanau, Germany). The pH was determined using a pH meter (Sartorius PT-21, Shanghai, China) and a soil-to-ddH_2_O ratio of 1:2.5. Using a flow injection autoanalyzer, nitrate (${\text{NO}}_{3}^{-}$-N) and ammonium (${\text{NH}}_{4}^{+}$-N) levels in sediment and water samples were determined (Westco Module 200, Smart Chem, France). Using an auto analyzer, the total phosphorus in sediment and water samples were also analyzed (AA3, Norderstedt, Germany). To evaluate the sediment moisture, 15 g of recent sediment was dehydrated in a 105 °C oven for 48 h. A TOC analyzer analyzed the total soluble organic carbon (TOC) in water samples (vario TOC cube, Elementar, Germany).

### 2.3. Sequencing using Illumina Miseq

Implementing the guidelines provided by the manufacturer, an Omega Soil DNA Kit was used to obtain the total DNA of the water and sediment (M5635-02, Omega Bio-Tek, Norcross, GA, USA). In order to amplify the bacteria's V3-V4 hypervariable region sequences, the primers 338F (ACTCCTACGGGAGGCAGCAG) and 806R were used (GGACTACHVGGGTWTCTAAT). The MiSeq system performed pair-end sequencing with the Illumina MiSeq Reagent Kit v3 in Shanghai Personal Biotechnology Co., Ltd. (Shanghai, China). Illumina MiSeq-PE300 was used for all sequencing operations (Illumina, San Diego, CA, USA). Bioinformatics of the microbiota was performed using QIIME 2 (Bolyen et al., 2019). After the unprocessed measurements with paired ends had been demultiplexed using demux plugin, the primers were cut using the cut adapt plugin (Martin, 2011). DADA2 procedure was used to quality-filter and denoise the sequences during settings (Callahan et al., 2016). To control sequence quality and eliminate any sequencing problems, we used the default parameters for the learnErrors, derepFastq, dada, and mergePairs methods. Afterwards, the amplicon sequence variations (ASVs) of the singletons were discarded. Then, using the Silva v132 database (http://www.arb-silva.de), the taxonomic identities of bacteria were determined (Quast et al., 2013).

The BioProject numbers were included with raw sequencing information uploaded on the National Center for Biotechnology Information's Sequence Read Archive (SRA) database: PRJNA 898838 for the bacteria sequences.

### 2.4. Statistical analysis

We calculated observed species using indexes like Shannon, Chao1, the diversity of phylogeny (Faith's PD), Simpson, evenness (Pielou), and Good's coverage to evaluate Alpha-diversity variations among water and sediment. Differential patterns in the community composition were visualized using non-metric multidimensional scaling (NMDS). The comparison of similarities (ANOSIM) method was used to investigate how different bacterial communities existed in various groups. There are proven Spearman relationships between environmental variables and the variety and structure of bacteria in water and sediment. The effect size of linear discriminant analysis (LEfSe) was utilized to differentiate the abundant features of bacterial communities among groups.

The underlying mechanism(s) affecting the community assembly was identified by employing the average nearest taxon distance (MNTD) and the maximum pairwise phylogenetic distance (MPD) calculated (Stegen et al., 2012). The “comdistnt” function from the R package Picante was used to determine the abundance-weighted beta closest taxon index (NTI), which measures the variation in the mean of the random null expectation from the observed mean nearest taxon distance (MNTD) (999 for the number of randomizations) (Stegen et al., 2012). After that, the comparative impact of the assembly process and underlying processes (Variable selection, homogenous selection, dispersal limitation, dispersal homogenizing, and undominated) were assessed using the NTI and modified Raup-Crick (RC_Bray_) metrics (Chase et al., 2011; Stegen et al., 2015).

Moreover, co-occurrence networks of water, surface, and subsurface sediments were constructed. The core microbial amplicon sequence variants (ASVs) shared by more than 60% of samples and with a mean relative abundance of more than 0.1% were chosen to minimize the effects of site-specific ASVs upon the network layout. SparCC was then employed to create the co-occurrence networks. Nodes using a direct correlation value larger than 0.6 and a *p*-value lower than 0.01 were chosen to form the network. In order to see the network, Cytoscape 3.8.2 was used (Shannon et al., 2003). The degree of centrality, proximity of centrality, number of vertices and edges, and betweenness of centrality and modularity are a few examples of known metrics that were used to define the topology of the networks. Natural connectedness after “attacking” edges or nodes can be used to gauge how robust a network is: higher resilience to threats to natural connectivity are signs of a more strong or stable network (Wu et al., 2021).

The contributions of the designated source sites to the bacterial community composition of the designated sink were predicted with the Bayesian classifier software program SourceTracker26 using R package SourceTracker v1.0.1. The designated sink and source sites' precise configurations are shown in Supplementary Table 1. The ASV tables and files produced by quality control were utilized. It has been shown that Source Tracker analysis with default settings, which include a rarefaction Alpha (0.001), beta (0.01), and depth of 1,000. Provide relatively good sensitivity, specificity, reliability, and precision. Five separate Source-Tracker runs were used to calculate the mean contribution proportion and variance for each source to identify and prevent any potential false positives. The relative standard deviation (RSD), which is used to calculate the belief in the mean, was used to anticipate and utilize the ratio of averaged expected percentage. Source proportions were computed: Statistical evaluations were performed using the, stated R environment (v3.5.1; http://www.r-project.org/), unless otherwise noted.

## 3. Results

### 3.1. Co-existing water and sediment microorganisms have diverse community structures

**Figure 2:**
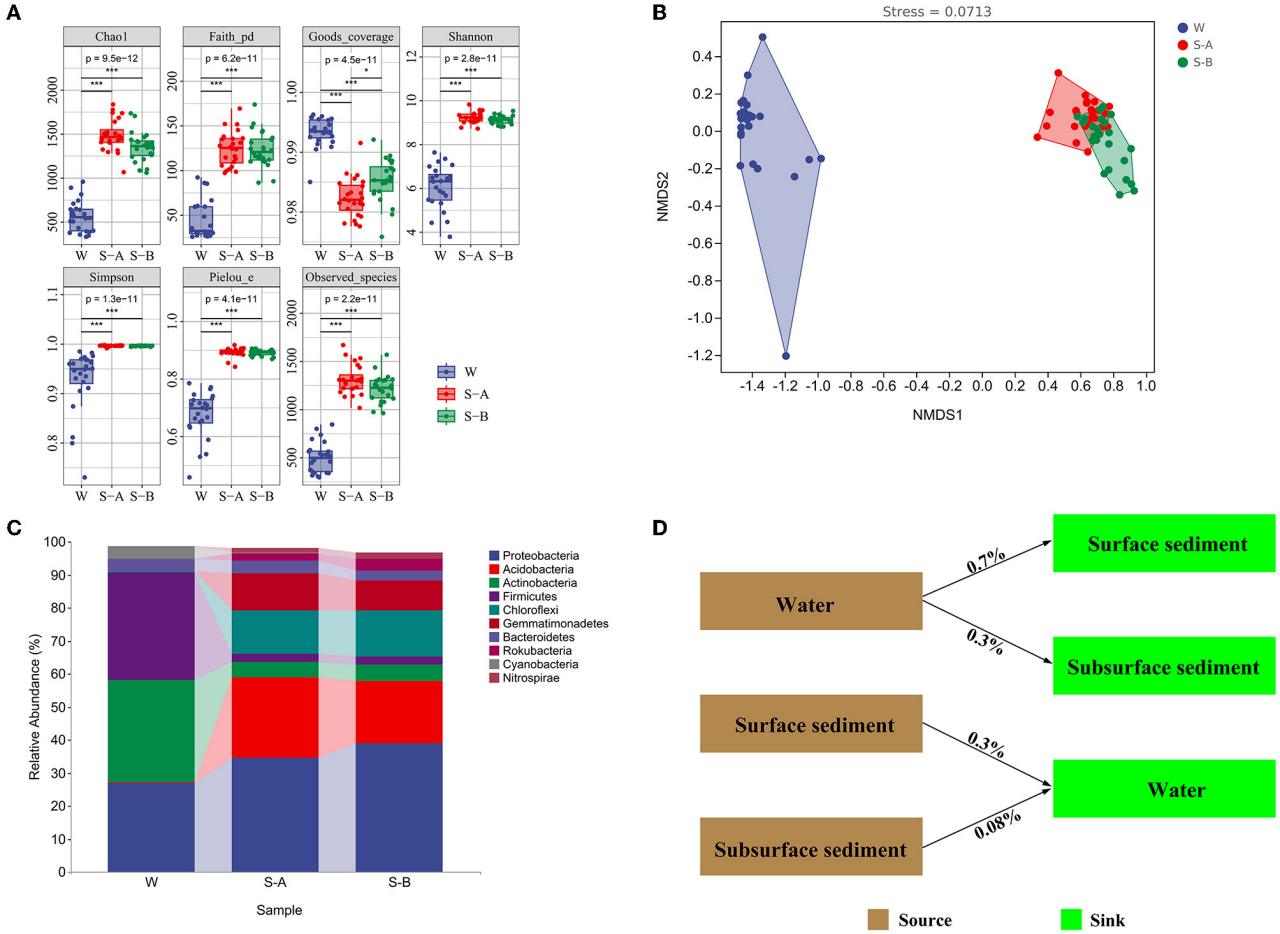
Bacterial composition, diversity, and community structure of water and sediment. **(A)** The alpha diversity of bacterial communities in the water and sediment. **(B)** The non-metric multidimensional scaling plots for the bacterial community structure of water and sediment. **(C)** The composition of bacteria at the phylum level. **(D)** SourceTracker analysis showing the potential sources of bacterial communities in water and sediment. W, water; S-A, surface sediments; S-B, subsurface sediment. *P < 0.05, **P < 0.01, ***P < 0.001.

The bacterial communities' alpha diversity within water and sediment is shown in Figure 2A. In water, the Chao1 index was between 333 and 959, the PD ranged from 25 to 92, and the Shannon index ranged between 3.7 and 7.6. The Shannon index varied between 8.7 and 9.8, the PD varied from 97 to 169, and the Chao1 index varied from 1,068 to 1,777 in surface sediment. The Shannon index was between 8.8 and 9.5, the PD varied from 86 to 173, and the Chao1 index ranged from 1,061 to 1,737 in subsurface sediment.

Bacterial community compositions in the water and sediment samples varied significantly, according to NMDS ordinations and comparison of similarity (ANOSIM) (*P* < 0.001; Figure 2B; Supplementary Table 2). There was a significant structural difference between the surface and subsurface sediment bacterial communities (Figure 2B). In sediment, the phyla Proteobacteria, Acidobacteria, and Chloroflexi have larger relative abundances (34.6–38.8%, 18.9–24.3%, and 13.2–13.9%, respectively) than in water (27, 0, and 0.3%, respectively). However, compared to sediment (2.3–4.6% and 2.4–4.8%, respectively), the relative amounts of Actinobacteria and Firmicutes were higher in water (30.9 and 36.2%Figure 2C). The Source Tracker results revealed that only 0.3% bacterial ASVs inside water came from the surface sediment and 0.08% from the subsurface sediment. Water is the source of 0.7% of bacterial ASVs in the surface sediment and 0.3% of the bacterial ASVs in the subsurface sediment (Figure 2D).

**Figure 3:**
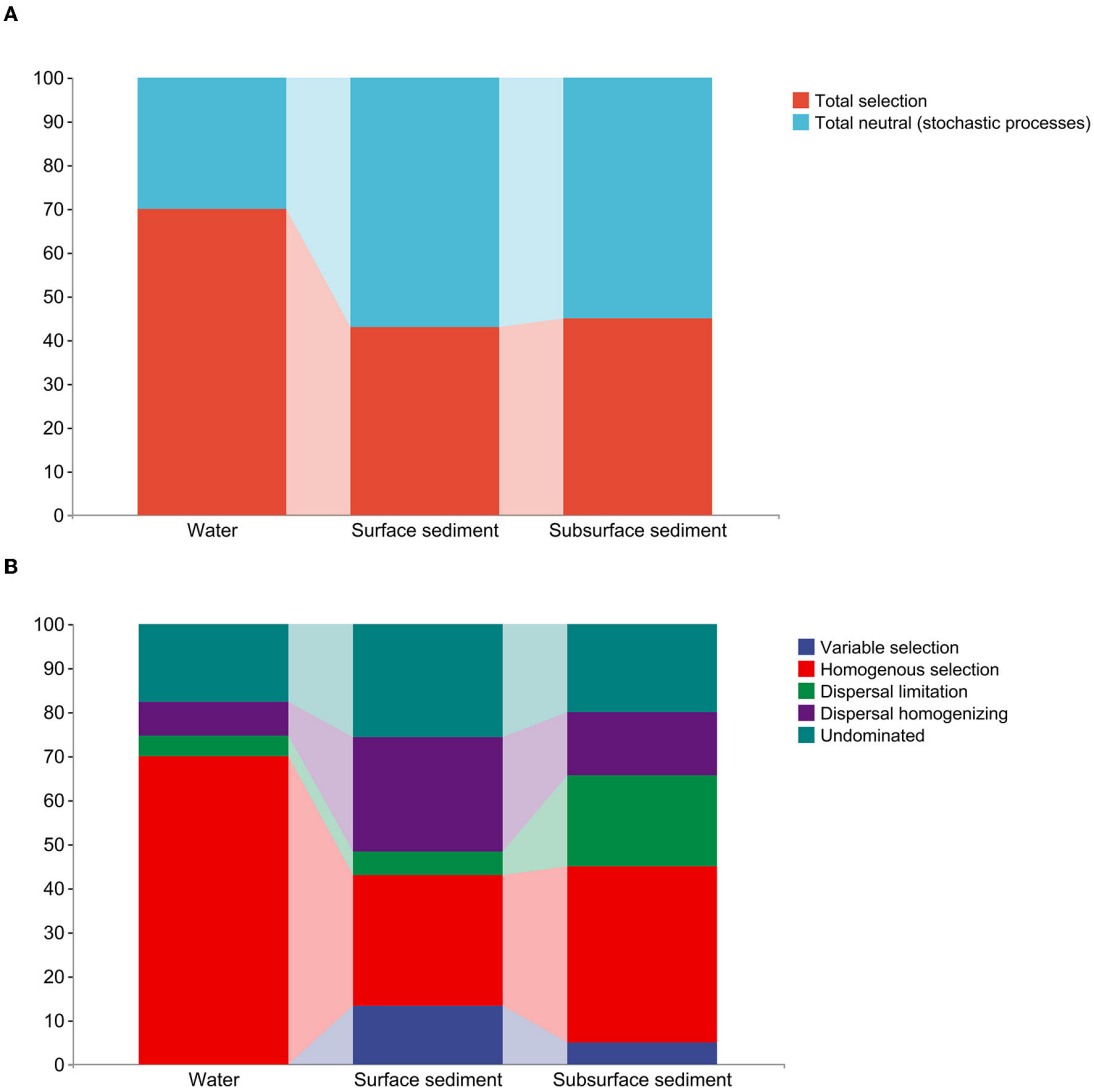
The quantitative estimate of the stochastic and deterministic processes for bacterial community assembly in the water and sediment. **(A)** The relative contribution (%) of determinism and stochasticity on the bacterial community assembly based on the β-Nearest Taxon Index (βNTI) values. **(B)** The relative contribution (%) of five ecological processes in the water and sediment.

The examination of the null model found that deterministic processes took control of the bacterial communities in the water, whereas bacterial populations in the sediment were largely dominated by stochastic processes (Figure 3, Supplementary Table 3). Homogenous selection played a major role in controlling how bacterial colonies gathered in the water. The bacterial community in surface sediment assembly mainly comprises homogenous selection, dispersal homogenizing, and undominated processes (weak selection, weak dispersal, diversification, and/or drift). In subsurface sediment assembly, the bacterial community is primarily characterized by homogenous selection, dispersal limiting, dispersal homogenizing, and undominated processes.

**Figure 4:**
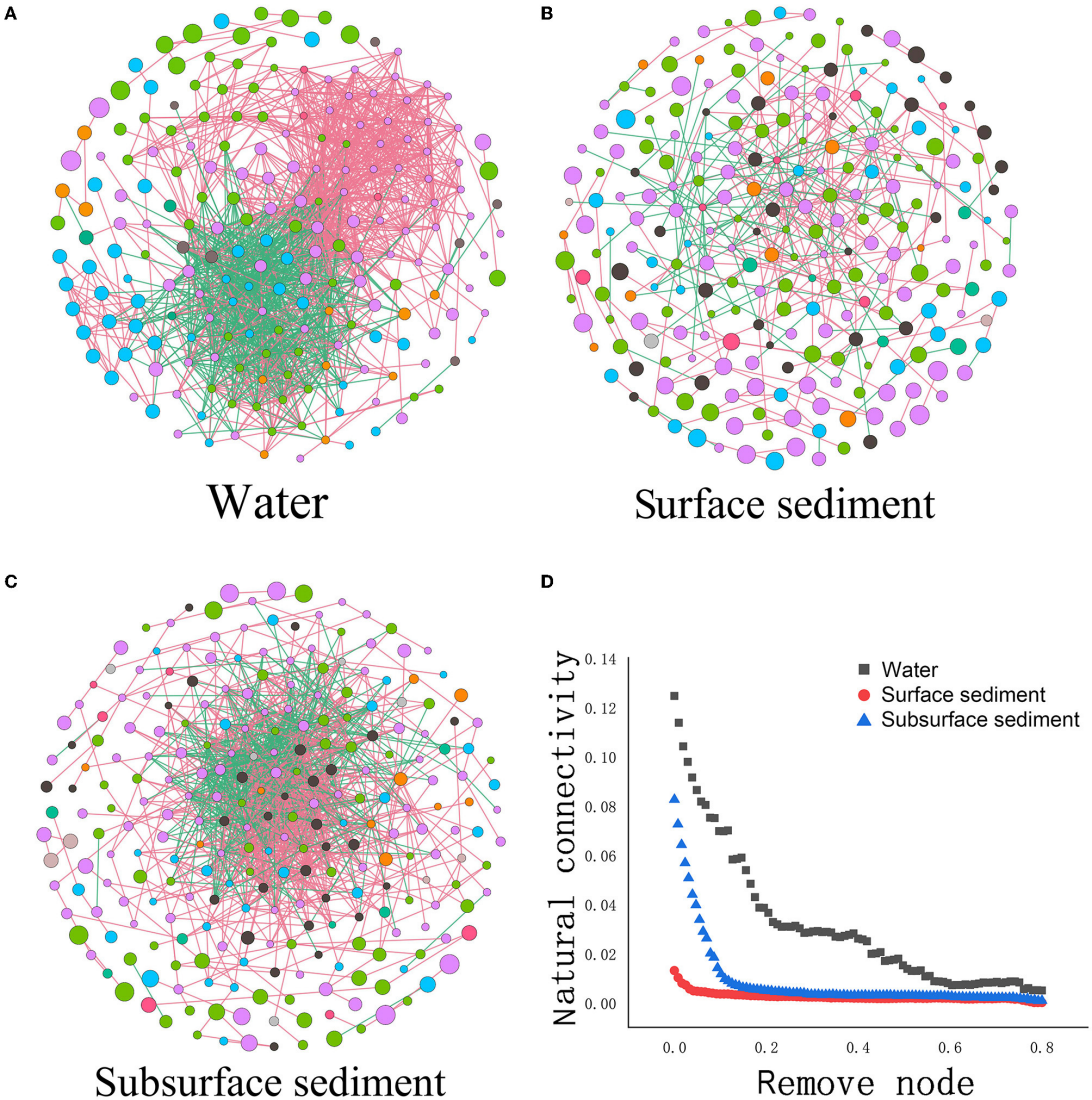
Microbial networks **(A–C)** and the network stability **(D)** in the water and sediment [Each node represents an ASV, and the edges connecting nodes represent either positive (green) or negative (red) correlations inferred using the SparCC approach using observed taxonomic units (OTU) abundance profiles (pseudo *P* < 0.05, correlation values < −0.6 or >0.6)].

Analysis of the co-occurrence network revealed that the number of nodes in the water was less than that in sediment, but the number of edges/nodes was greater than that in sediment (Figure 4 and Supplementary Table 4). The average number of neighbors, clustering coefficients, network density, network centralization, and the negative correlation ratio in water were also higher than those in sediment. We also performed a resilience test to gauge a network's resistance to node or edge attacks through changes in connectivity that occur naturally. We found that the natural connectivity of the water network reduced more significantly and fluctuated in severity more than sediment by deleting the identical number of nodes or edges, indicating weakened resistance. Natural connectivity based on robustness tests also revealed that surface sediment was more resistant to node or edge attack than subsurface sediment.

### 3.2. Relationships between environmental and bacterial community

Among the environmental parameters for water, the ${\text{NO}}_{3}^{-}$-N demonstrated the most positive associations with Cyanobacteria, Chloroflexi, Planctomycetes, and the Chao1 index, and the most negative associations with Firmicutes (Supplementary Figure 1). The ${\text{NH}}_{4}^{+}$-N demonstrated the most positive associations with Acidobacteria, Verrucomicrobia, and the Shannon index. In surface sediment samples, however, the ${\text{NO}}_{3}^{-}$-N was most significantly connected with positive Acidobacteria, Gemmatimonadetes, and the Simpson index, and most negatively correlated with Actinobacteria and Firmicutes. The ${\text{NH}}_{4}^{+}$-N had the most powerfully positive correlation with Proteobacteria, and the most negative correlations with Firmicutes and Rokubacteria. TN had the most significant negative correlations with Chloroflexi. In subsurface sediment samples, the ${\text{NH}}_{4}^{+}$-N was most significantly connected with positivity with Acidobacteria, and most strongly inversely associated with Chloroflexi. The Simpson index and the TP had the strongest positive correlations, whereas the pH had the strongest positive correlation with Nitrospirae.

### 3.3. The microorganisms in sediment are motivated by contrasting plant communities

**Figure 5:**
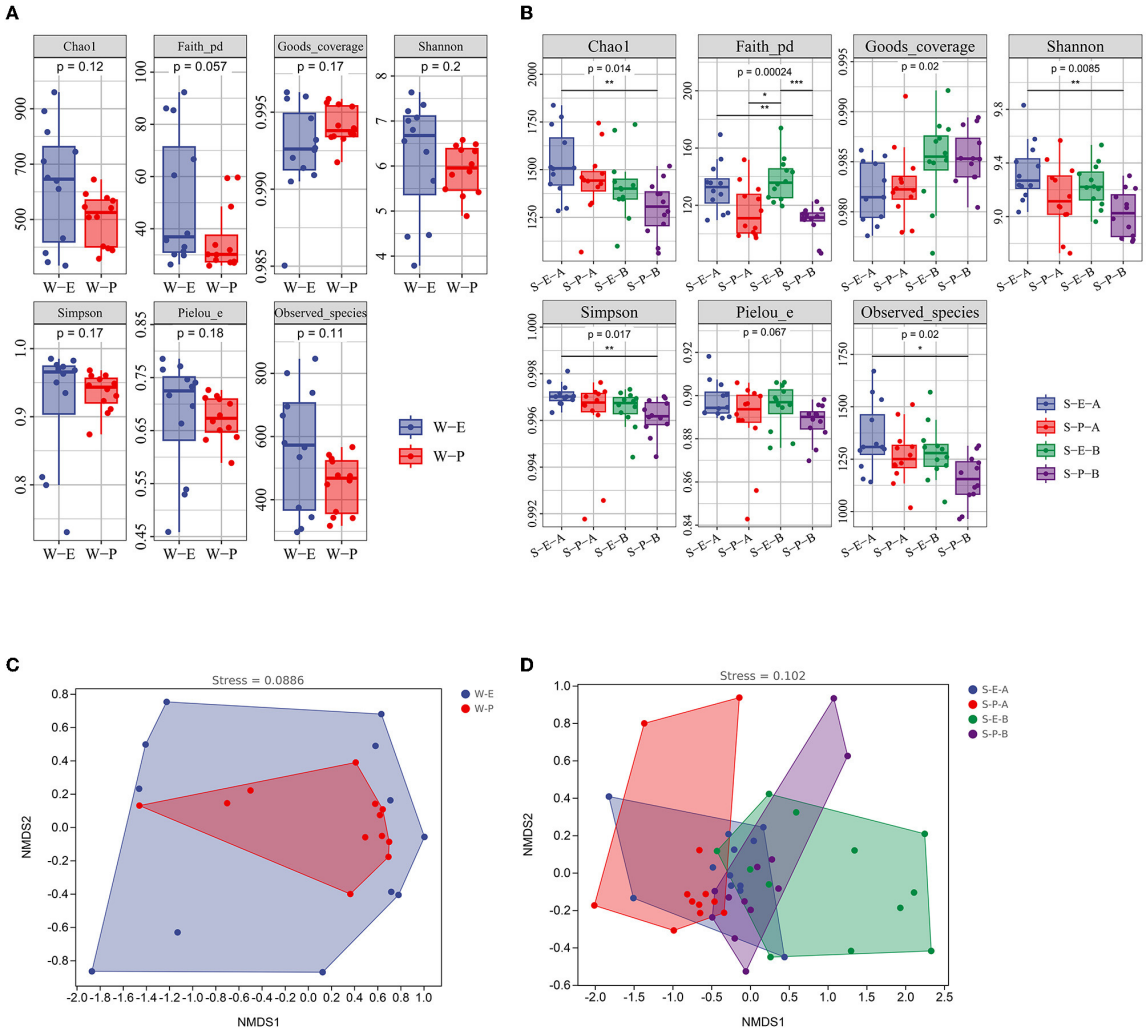
Alpha diversity indices **(A, B)** and non-metric multidimensional scaling plots **(C, D)** of bacterial communities in different plants. S-E-A, the surface sediment of *Erigeron canadensis* L; S-E-B, the subsurface sediment of Erigeron canadensis L; S-P-A, the surface sediment of *Phragmites australis* (Cav.) Trin. Ex Steud; S-P-B, the subsurface sediment of *Phragmites australis* (Cav.) Trin. Ex Steud. Significance: ^*^P < 0.05, ^**^P < 0.01, ^***^P < 0.001.

In the sediments (Surface and subsurface), a significant difference was found between the *Erigeron canadensis* L and *Phragmites australis* (Cav.) Trin. Ex Steud (Figure 5). Phragmites australis (Cav.) Trin. Ex Steud's bacterial community was indexed by Chao1, Shannon, and Simpson, which were significantly lower than those of *Erigeron canadensis* L in both the surface and subsurface sediment. The results of the ANOSIM test and NMDS analysis revealed no significant correlation. While a considerable variation was discovered between both the surface and subsurface sediment of the two plants, there were no differences in the bacterial population in the water between the two plants (Figure 5 and Supplementary Table 5).

Significant variation in bacterial community composition was detected between different plant communities (*p* < 0.001) (Supplementary Figure 2). The relative abundance of *Subgroup7, Subgroup6, Anaerolinea, AKYG1722, Gitt GS136, C0119, Gemmatimonas, Sphingomonas, Ramlibacter*, and *Ellin6067* in the surface sediment of Australian Phragmites (Cav.) was found. Compared to the other villages, Trin. Ex Steud was much higher (*p* < 0.01). The proportional prevalence of *PAUC26f* , *Subgroup 22, Subgroup 9, A4b, S085, Desulfuromonas, MBNT15, Azoarcus, PLTA13, Pseudomonas*, and *Rokubacteriales* in the subsurface sediment of *Erigeron canadensis* L was substantially higher than that in the *Phragmites australis* subsurface silt (Cav.) Trin. Ex Steud. The relative abundance of *Latescibacteria, Haliangium*, and *MND1* in the subsurface sediment of *Phragmites australis* (Cav.) Trin. Ex Steud was higher than that of other communities.

### 3.4. The bacterial community of water shows dynamic temporal shifts in patterns

**Figure 6:**
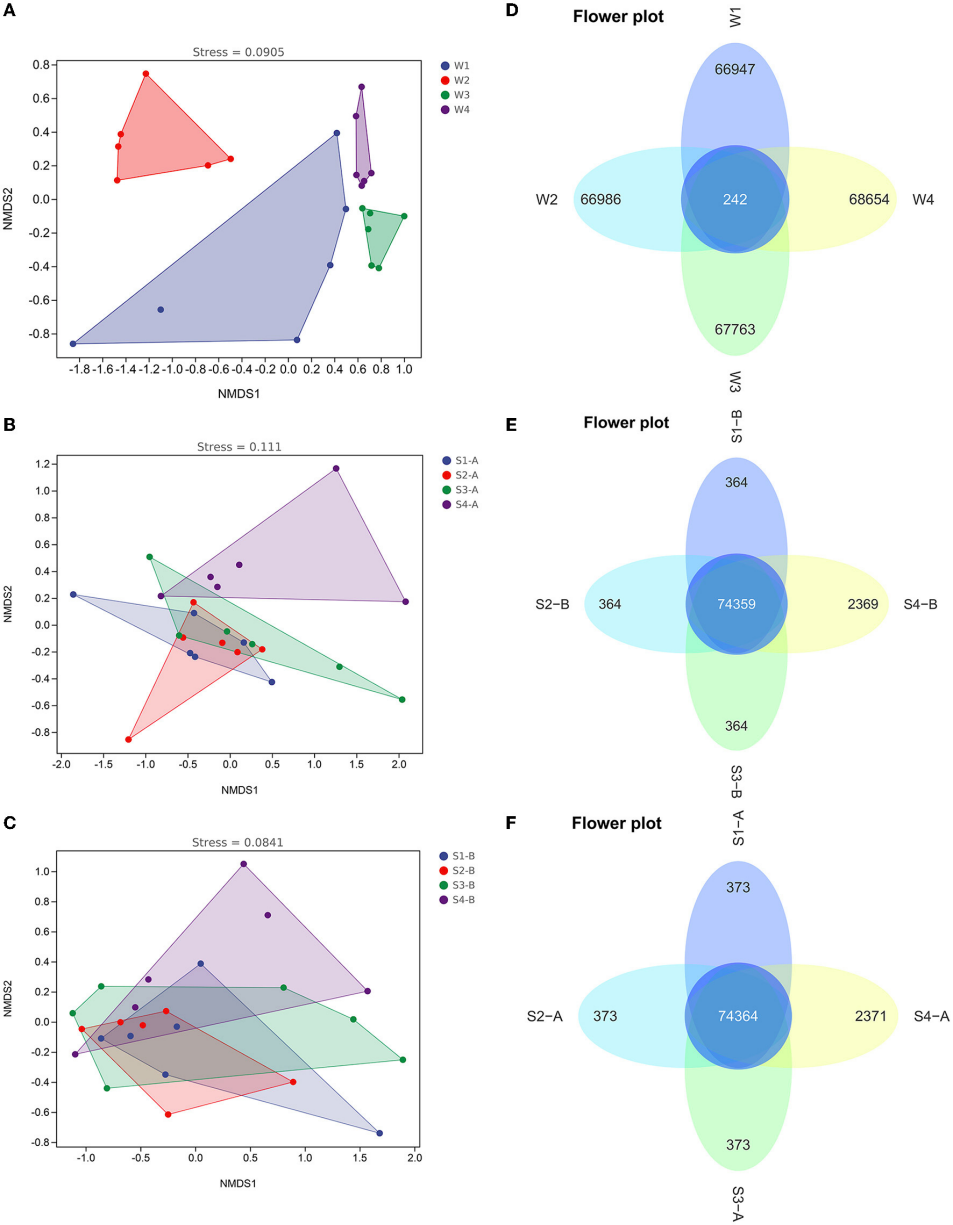
The non-metric multidimensional scaling plots **(A–C)** and Venn diagrams **(D–F)** in the bacterial communities of water and sediment between the four times samples. W1, water sampled for the first time; W2, water sampled for the second time; W3, water sampled for the third time; W4, water sampled for the fourth time; S1, sediment sampled for the first time; S2, sediment sampled for the second time; S3, sediment sampled for the third time; S4, sediment sampled for the fourth time; A, surface sediment; B, subsurface sediment.

The alpha diversity of the fourth water sample shows a significant difference, although there is no significant difference in the samples of surface and subsurface sediments (Supplementary Figure 3). The Chao1, Simpson, and Shannon indexes among the four samples showed the number of microbiological colonies was the highest in the second water sample and lowest in the fourth. Results of the NMDS analysis and the ANOSIM test showed that there were notable variations in the bacterial communities of water between the four times samples (Figure 6 and Supplementary Table 6), while there was no significant difference in the bacterial communities of the surface and subsurface sediments between the four times samples (Figure 6 and Supplementary Table 7).

Most of the microorganisms in the communities were Firmicutes, Actinobacteria, Bacteroidetes, Proteobacteria, and Cyanobacteria in water (Supplementary Figure 4). We discovered that the abundance of these dominant bacteria fluctuated in the four samples we collected. The results of Source Tracker revealed that 52% of the ASVs in the water sampled for the second time came from the water sampled for the first time, 44% of the ASVs in the water sampled for the third time came from the water sampled for the second time, and 91% of the ASVs in the water sampled for the fourth time came from the water sampled for the third time (Supplementary Figure 5). The majority of the microorganisms in the communities were Proteobacteria, Acidobacteria, Chloroflexi, Gemmatimonadetes, the bacterial genus Bacteroidetes, and Firmicutes in surface and subsurface sediments, and the relative abundance in the sediments sampled four times is relatively stable (Supplementary Figure 6).

LEfSe revealed that the abundance of Alsobacter in the first water sample compared to the other three periods was considerably higher. However, the abundance of Acidobacteria, Chloroflexi, Cyanobacteria, and Verrucomicrobia in the second water sample, compared to the other groups, was substantially higher (Supplementary Figure 7). In comparison to the other three water samples, the fourth had a much higher number of Actinobacteria. The Venn diagram showed that there are only 242 common ASVs in the fourth water sample, accounting for 0.3% of the total ASV, while there are 74,364 and 74,359 common ASVs in the surface and subsurface sediments of the fourth water sample, accounting for 96.8 and 96.8%, respectively (Figure 6).

The null model analysis revealed that the third and fourth water samples had greater deterministic bacteria community formation processes than the first and second water samples (Supplementary Table 8).

## 4. Discussion

In this research, we thoroughly investigated the symbiotic water and sediment bacterial diversity, community composition, and co-occurrence patterns of various time dynamics and plant communities of the Yellow River floodplain ecosystem. The sediment bacterial community had a substantially higher level of α-diversity than water. We found that bacterial communities were significantly influenced by water and sediment, with low microbial interactions in co-existing water and sediment communities. Furthermore, bacterial community structure varied between surface and subsurface sediments. Moreover, the depth and various plant covers of the floodplain ecosystem impacted the bacterial community structure of the sediment. The water environment was selected for particular communities of microbes that come together gradually, in an unpredictable and non-random manner, whereas the sediment environment was relatively stable, and the bacterial communities gathered randomly. The sediment was more robust to node or edge attack than the water. These results expand our knowledge of microbes in co-existing water and sediment communities.

### 4.1. A limited overlap of bacterial communities in interactions between water and sediment

Studies have shown that sediments can act as reservoirs, absorbing and subsequently depositing microorganisms from surrounding areas, such as soil and plant detritus (Crump et al., 2012). In addition, sediments are formed over a long period through soil deposition and erosion. In this process, the selective transport of light particles and labile organic components occurs (Mueller-nedebock et al., 2016). Deposition increases moisture and nutrient content simultaneously and may change pH (Berhe and Kleber, 2013). These modifications made in sedimentary environments may greatly enhance bacterial diversity. According to the Spearman correlations analysis, many environmental conditions impact bacteria co-existing in sediment and water. The bacterial communities clearly distinguished between the habitations in the water and the communities in the sediment, indicating that the habitat was primarily responsible for the differences in community structure. The results of SourceTracker also show that the bacterial ASVs in water were rarely from sediment, and the bacterial ASVs in sediment were rarely from water, which suggested that their interactions were minimal.

In addition, we believe that the difference in community assembly among different habitats is also a factor that will cause variations in the composition of bacterial communities. While stochastic mechanisms dominated the bacterial communities in the sediment, the bacterial species in the water were dominated by deterministic mechanisms. The non-random, niche-based mechanisms that underlie the deterministic process include environmental filtering and numerous biological interactions (Zhou and Ning, 2017). Stochastic process refers to the random change of community structure caused by birth, death, immigration, and/or historical contingencies (Zhou and Ning, 2017). In general, the process of deterministic assembly corresponds to low nutrient conditions, and large environmental changes lead to deterministic assembly (Yu et al., 2019). The stochastic assembly is more likely to occur in small habitats with abundant species, high production, little disturbance, and/or little predation. Due to the elimination of certain functions, the deterministic process predominates in low-diversity communities while the stochastic process predominates in communities with a high level of diversity (Xun et al., 2019).

The current study proved that the network's complexity increased in water compared to sediment. We also discovered that water strengthened the network's members' negative correlation. The rise in the negative correlation ratio indicated increased competition between soil microbial communities. We also found that compared to sediment, the bacterial network in water exhibits unstable properties. Earlier research has revealed that improved node connection (Fan et al., 2018), centrality (Jordán, 2009), and complexity (Schoener, 1974) are linked to diminished network stability. According to the robustness assessments based on natural connection, compared with sediment, the robustness of the bacteria network in the water against node or edge attacks was reduced.

### 4.2. Soil depth significantly affected the bacterial community structure of the sediment

Previous studies have shown that the soil properties of the Yellow River floodplain changed significantly with soil depth (Yu et al., 2020; Chen et al., 2022). The main reason for the difference in depth may be that the surface soil is greatly affected by the input of root exudates and plant litter, while the nutrient source of the subsurface soil is mainly the decomposition of dead roots (Jobbagy and Jackson, 2000). Wetland is impacted by long-term flooding, and the root biomass is the largest in the surface soil (Zeng et al., 2016). The difference between the surface and subsurface sediment environments also profoundly affects the bacterial community structure. Moreover, we also found that, compared with the surface sediment, the robustness of the bacterial network in the subsurface sediment was also reduced. As a result, the bacterial population in the surface sediment is more resistant to environmental change than the community in the deeper soil layers.

### 4.3. The bacterial communities of sediment are sensitive to plant types

In addition, we also noticed the different responses of bacterial communities to plant communities in water and sediment. The plant community in the floodplain ecosystem significantly affected the bacterial diversity and structure communities in sediment (surface and subsurface), but did not affect the structure of the bacterial community in water. Some studies throughout the Yellow River Delta found that microbial metabolic activities were closely related to plant covers, and the β-diversity patterns and function of the group of bacteria differed significantly between soils with different plant covers (Cao et al., 2019; Wei et al., 2020). In order to control growth conditions, by secreting bioactive compounds into the rhizosphere and changing the soil environment, plants can affect the soil microbiota (Hu et al., 2018).

### 4.4. Different temporal shifts patterns of bacteria in co-existing water and sediment

The patterns of the water's bacterial community exhibit dynamic temporal alterations. The results of Source Tracker analysis on the source of bacteria ASVs in all four water samples revealed that the bacteria ASVs in water fluctuated with sampling time. Venn diagrams indicated that the proportion of unique ASVs of bacterial communities in water at different sampling times was high, and the proportions of shared ASVs were low, whereas the opposite was true in sediment. This shows that the water at different times has a unique bacterial community composition and the water environment continuously changes. Our results indicate that the water selects particular assemblages of microorganisms that do not happen randomly or occur in a predictable way over time, while the sediment environment is relatively stable and the bacterial communities gather randomly. The research area was in the center and bottom of the Yellow River floodplain, and its hydrodynamic conditions are complex. During the two adjacent sampling times, due to the influence of the river flow discharge power, the water flow at the sampling location exchanged violently, resulting in significant differences in the bacterial communities of the different water samplings. In comparison to water, the exchange rate of sediment was slow, and the bacterial community was relatively stable.

## 5. Conclusions

This study investigated the diversity, community composition, and co-occurrence patterns of symbiotic bacteria in the water and sediment of different time dynamics and plant communities of the Yellow River floodplain ecosystem. The α-diversity of the bacterial community was substantially greater in sediment than in the water. The composition of the bacterial community in sediment and water differed greatly, and communication between them was very low. The aqueous environment attracts particular microbe groupings that come together gradually, in an unpredictable and non-random manner, whereas the sediment environment was relatively stable, and the bacterial communities gathered randomly. The sediment was more robust to node or edge attack than the water. The different plant covers and sediment depths of the floodplain ecosystem drastically changed the sediment's bacterial community structure. Understanding bacterial populations in sand and water that coexist gives a solid reference value for the service function and barrier effect of the floodplain ecosystem since microbial diversity, composition, and structure impact ecosystem stability.

## Data availability statement

The datasets presented in this study can be found in online repositories. The names of the repository/repositories and accession number(s) can be found in the article/Supplementary material.

## Author contributions

YY, YS, and JZ conceived the presented idea and received important feedback from all co-authors. Field management and soil chemical analysis was carried out by CW and BL. Sampling collections and DNA preparation were carried out by YY and ZH. MiSeq sequencing process and data analysis were carried by YY and NH. The manuscript was written by ZH and CW with help from YS, NH, YY, and JZ. All authors contributed to the article and approved the submitted version.

## References

[B1] Allison M. A. Kuehl S. A. Martin T. C. Hassan A. (1998). Importance of flood-plain sedimentation for river sediment budgets and terrigenous input to the oceans: insights from the Brahmaputra-Jamuna River. Geology. 26, 175–178. 10.1130/0091-7613(1998)026<0175:IOFPSF>2.3.CO;2

[B2] Altermatt F. (2013). Diversity in riverine metacommunities: a network perspective. Aquat. Ecol. 47, 365–377. 10.1007/s10452-013-9450-3

[B3] Berhe A. A. Kleber M. (2013). Erosion, deposition, and the persistence of soil organic matter: mechanistic considerations and problems with terminology. Earth Surf. Proc. Land. 38, 908–912. 10.1002/esp.3408

[B4] Bolyen E. Rideout J. R. Dillon M. R. Bokulich N. A. Abnet C. C. Al-Ghalith G. A. (2019). Reproducible, interactive, scalable and extensible microbiome data science using QIIME 2. Nat. Biotechnol. 9, 37. 10.1038/s41587-019-0209-9 31341288 PMC7015180

[B5] Callahan B. J. McMurdie P. J. Rosen M. J. Han A. W. Johnson A. J. A. Holmes S. P. (2016). DADA2: high-resolution sample inference from Illumina amplicon data. Nat. Methods. 13, 581–583. 10.1038/nmeth.3869 27214047 PMC4927377

[B6] Cao F. Q. Kong Q. Chen H. Y. Xu F. Zhao C. (2019). The structural characteristics of soil microbial composition in different wetland plants in the yellow river delta. Chiang Mai J. Sci. 46, 812–821. Available online at: http://epg.science.cmu.ac.th/ejournal/

[B7] Chase J. M. Kraft N. J. B. Smith K. G. Vellend M. Inouye B. D. (2011). Using null models to disentangle variation in community dissimilarity from variation in α-diversity. Ecosphere. 2, 1–11. 10.1890/ES10-00117.133092668

[B8] Chen Z. J. Xiao Y. T. Dong X. D. (2022). Effect of sediment deposition on soil stoichiometric ratios in the middle and lower section of the Yellow River. Chin. J. Ecol. 41, 8. 10.13292/j.1000-4890.202206.006

[B9] Cloutier D. D. Alm E. W. Mclellan S. L. Wommack K. E. (2015). Influence of land use, nutrients, and geography on microbial communities and fecal indicator abundance at lake michigan beaches. Appl. Environ. Microb. 81, 4904–4913. 10.1128/AEM.00233-15 25979888 PMC4495187

[B10] Costanza R. Groot R. D. Sutton P. Ploeg S. Anderson S. J. Kubiszewski I.. (2014). Changes in the global value of ecosystem services. Global Environ. Chang. 26,152–158. 10.1016/j.gloenvcha.2014.04.002

[B11] Crump B. C. Amaral-zettler L. A. Kling G. W. (2012). Microbial diversity in arcticfreshwaters is structured by inoculation of microbes from Soils. ISME J. 6, 629–1639. 10.1038/ismej.2012.9 22378536 PMC3498914

[B12] Du L. Wang R. Gao X. Hu Y. Guo S. (2020). Divergent responses of soil bacterial communities in erosion-deposition plots on the loess plateau. Geoderma. 358, 113995. 10.1016/j.geoderma.2019.113995

[B13] Fan K. K. Weisenhornc P. Gilbertc J. A. Chua H. Y. (2018). Wheat rhizosphere harbors a less complex and more stable microbial co- occurrence pattern than bulk soil. Soil Biol. Biochem. 125, 251–260. 10.1016/j.soilbio.2018.07.022

[B14] Fan Z. Han R. M. Ma J. Wang G. X. (2016). Submerged macrophytes shape the abundance and diversity of bacterial denitrifiers in bacterioplankton and epiphyton in the Shallow Fresh Lake Taihu, China. Environ. Sci. Pollut. Res. Int. 23, 14102–14114. 10.1007/s11356-016-6390-1 27048324

[B15] Fillinger L. Zhou Y. Kellermann C. Griebler C. (2019). Non-random processes determine the colonization of groundwater sediments by microbial communities in a pristine porous aquifer. Environ. Microbiol. 21, 327–342. 10.1111/1462-2920.14463 30378251

[B16] Hu L. F. Robert C. A. M. Cadot S. Zhang X. Ye M. Li B.. (2018). Root exudate metabolites drive plant-soil feedbacks on growth and defense by shaping the rhizosphere microbiota. Nat. Commun. 9, 2738. 10.1038/s41467-018-05122-7 30013066 PMC6048113

[B17] Jiao C. Zhao D. Zeng J. Guo L. Yu Z. (2020). Disentangling the seasonal co-occurrence patterns and ecological stochasticity of planktonic and benthic bacterial communities within multiple lakes. Sci. Total Environ. 740, 140010. 10.1016/j.scitotenv.2020.140010 32563874

[B18] Jobbagy E. E. G. Jackson R. B. (2000). The vertical distribution of soil organic carbon and its relation to climate and plant. Ecol. Appl. 10, 423–436. 10.1890/1051-0761(2000)010[0423:TVDOSO]2.0.CO;2

[B19] Jordán F. (2009). Keystone species and food webs. Philos. T R Soc. B. 364, 1733–1741. 10.1098/rstb.2008.0335 19451124 PMC2685432

[B20] Li Y. He Q. K. Ma X. W. Wang H. J. Liu C. H. (2019). Plant traits interacting with sediment properties regulate sediment microbial composition under different aquatic DIC levels caused by rising atmospheric CO^2^. Plant Soil. 445, 497–512. 10.1007/s11104-019-04312-6

[B21] Mari L. Casagrandi R. Bertuzzo E. Rinaldo A. Gatto M. (2014). Metapopulation persistence and species spread in river networks. Ecol. Lett. 17, 426–434. 10.1111/ele.12242 24460729

[B22] Martin M. (2011). CUTADAPT removes adapter sequences from high-throughput sequencing reads. EMBnet J. 17, 10–12. 10.14806/ej.17.1.20028715235

[B23] Mueller-nedebock D. Chivenge P. Chaplot V. (2016). Selective organic carbon lossesfrom soils by sheet erosion and main controls. Earth Surf. Proc. Land. 41,1399–1408. 10.1002/esp.3916

[B24] Nevers M. B. Byappanahalli M. N. Nakatsu C. H. Kinzelman J. L. Phanikumar M. S. Shively D. A.. (2020). Interaction of bacterial communities and indicators of water quality in shoreline sand, sediment, and water of Lake Michigan. Water Res. 178, 115671. 10.1016/j.watres.2020.115671 32380294

[B25] Quast C. Pruesse E. Yilmaz P. Gerken J. Schweer T. Yarza P.. (2013). The SILVA ribosomal RNA gene database project: improved data processing and web-based tools. Nucl. Acids Res. 41, 590–596. 10.1093/nar/gks1219 23193283 PMC3531112

[B26] Schoener T. W. (1974). Stability and complexity in model ecosystems. Evolution. 28, 510–511. 10.1111/j.1558-5646.1974.tb00784.x

[B27] Shannon P. Markiel A. Ozier O. Baliga N. S. Wang J. T. Ramage D.. (2003). Cytoscape: a software environment for integrated models of biomolecular interaction networks. Genome Res. 13, 2498–2504. 10.1101/gr.1239303 14597658 PMC403769

[B28] Stegen J. C. Lin X. Fredrickson J. K. Konopka A. E. (2015). Estimating and mapping ecological processes influencing microbial community assembly. *Front*. Microbiol. 6, 370. 10.3389/fmicb.2015.00370 25983725 PMC4416444

[B29] Stegen J. C. Lin X. Konopka A. E. Fredrickson J. K. (2012). Stochastic and deterministic assembly processes in subsurface microbial communities. ISME J. 6, 1653–1664. 10.1038/ismej.2012.22 22456445 PMC3498916

[B30] Wang C. Liu S. Zhang Y. Liu B. He F. Xu D.. (2018). Bacterial communities and their predicted functions explain the sediment nitrogen changes along with submerged macrophyte restoration. Microb. Ecol. 76, 625–636. 10.1007/s00248-018-1166-4 29502133

[B31] Wei G. Li M. Shi W. Tian R. Chang C. Wang Z.. (2020). Similar drivers but different effects lead to distinct ecological patterns of soil bacterial and archaeal communities. *Soil Biol*. Biochem. 114,107759. 10.1016/j.soilbio.2020.107759

[B32] Wu M. H. Chen S. Y. Chen J. W. Xue K. Wang Y. F. (2021). Reduced microbial stability in the active layer is associated with carbon loss under alpine permafrost degradation. *Proc. Natl. Acad. Sci*. USA. 118, e2025321118. 10.1073/pnas.2025321118 34131077 PMC8237688

[B33] Xia N. Xia X. H. Liu T. Hu L. J. Zhu B. T. Zhang X. T.. (2014). Characteristics of bacterial community in the water and surface sediment of the yellow river, China, the largest turbid river in the world. J. Soil Sediment. 14, 1894–1904. 10.1007/s11368-014-0974-5

[B34] Xia P. Yan D. Sun R. Song X. Yi Y. (2020). Community composition and correlations between bacteria and algae within epiphytic biofilms on submerged macrophytes in a plateau lake, southwest china. Sci. Total Environt. 727,138398. 10.1016/j.scitotenv.2020.138398 32335447

[B35] Xun W. B. Li W. Xiong W. Ren Y. Liu Y. P. Miao Y. Z.. (2019). Diversity-triggered deterministic bacterial assembly constrains community functions. Nat. Commun. 10, 3833. 10.1038/s41467-019-11787-5 31444343 PMC6707308

[B36] Yu Y. J. Wu M. Petropoulos E. Zhang J. W. Nie J. Liao Y. L.. (2019). Responses of paddy soil bacterial community assembly to different long-term fertilizations in southeast China. Sci. Total Environt. 656, 625–633. 10.1016/j.scitotenv.2018.11.359 30529966

[B37] Yu Y. Y. Cui M. Xiao Y. T. Chang M. Y. Wang C. Zhao L.. (2020). Quantitative estimation of stochastic and deterministic processes for soil prokaryotic community assembly in the Yellow River floodplain. Eur. J. Soil Sci. 72, 1462–1477. 10.1111/ejss.13056

[B38] Zeng Q. C. Li X. Dong Y. H. An S. S. (2016). Ecological stoichiometry of soils in the yanhe watershed in the loess plateau: the influence of different plant zones. J. Nat. Resour. 31, 1881–1891. 10.11849/zrzyxb.20160038

[B39] Zhang L. Y. Manuel D. B. Shi Y. Liu X. Yang Y. F. Chu H. Y. (2021). Co-existing water and sediment bacteria are driven by contrasting environmental factors across glacier-fed aquatic systems. Water Res. 198, 117139. 10.1016/j.watres.2021.117139 33895591

[B40] Zhang M. Huang H. Q. Zhang X. H. (2016). A study of the characteristics of sedimentation in the Lower Yellow River during overbank floods. Adv. Water Sci. 27, 165–175. 10.14042/j.cnki.32.1309.2016.02.001

[B41] Zhang X. Yan Q. Y. Yu Y. H. Dai L. L. (2014). Spatiotemporal pattern of bacterioplankton in Donghu Lake. Chin. J. Oceanol. Limn. 32, 554–564. 10.1007/s00343-014-3037-235760113

[B42] Zhou J. Ning D. (2017). Stochastic community assembly: does it matter in microbial ecology? Microbiol. Mol. Biol. R. 81, e00002–00017. 10.1128/MMBR.00002-17 29021219 PMC5706748

