# Supplementary material for: A limited overlap of interactions between the bacterial community of water and sediment in wetland ecosystem of the Yellow River floodplain
Source: Front Microbiol. 2023 Jun 22;14:1193940. doi: 10.3389/fmicb.2023.1193940 (PMC10325576; doi:10.3389/fmicb.2023.1193940)
Supplement: Supplementary file 1 [file Data_Sheet_1.docx]

**Supporting Information**

**Title:** A limited overlap of interactions between water and sediment in wetland ecosystem of the Yellow River floodplain

Zhiguang Han^1,2,5^, Cong Wang^1,4,5^, Binghai Lei^1,4^, Nan Hui^3^, Yanyan Yu^1,4*^, Yu Shi^1,4*^, Junqiang Zheng^1,^^4*^

^1^Yellow River Floodplain Ecosystems Research Station, School of Life Sciences, Henan University, Kaifeng, Henan, 475004, China.

^2^Department of Civil Engineering and Architecture, Henan University, Kaifeng, Henan, 475004, China.

^3^School of Agriculture and Biology, Shanghai Jiao Tong University, 800 Dongchuan Rd., 200240 Shanghai, China

^4^International Joint Research Laboratory for Global Change Ecology, School of Life Sciences, Henan University, Kaifeng, Henan, 475004, China.

^5^These authors contributed equally: Zhiguang Han and Cong Wang

******Corresponding authors*:** Yanyan Yu, Yu Shi and Junqiang Zheng

**E-mails:** Yanyan Yu: [463398083@qq.com](mailto:463398083@qq.com); Junqiang Zheng: [jqzheng@henu.edu.cn](mailto:jqzheng@henu.edu.cn); Yu Shi: [yshi@henu.edu.cn](mailto:yshi@henu.edu.cn)

**This file includes:**

**Fig S1 to S7**

**Table S1 to S8**

**
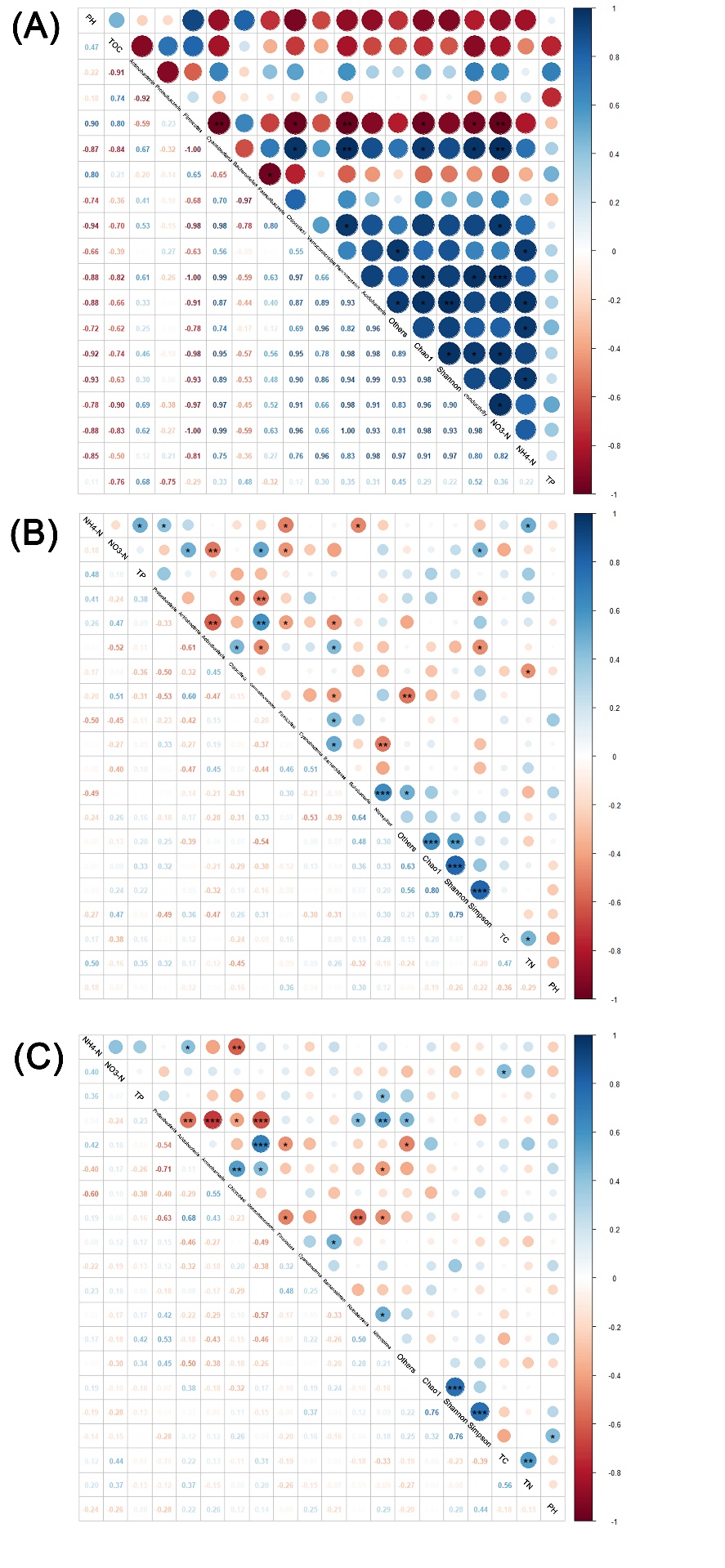
**

**Figure S1** Spearman correlations between environmental factors and bacterial diversity and composition in water and sediment. NO_3_^-^-N, nitrate nitrogen; NH_4_^+^-N, ammonium nitrogen; TC, total carbon; TN, total nitrogen; TP, total phosphorus. ∗P < 0.05, ∗∗P < 0.01, ∗∗∗P < 0.001.


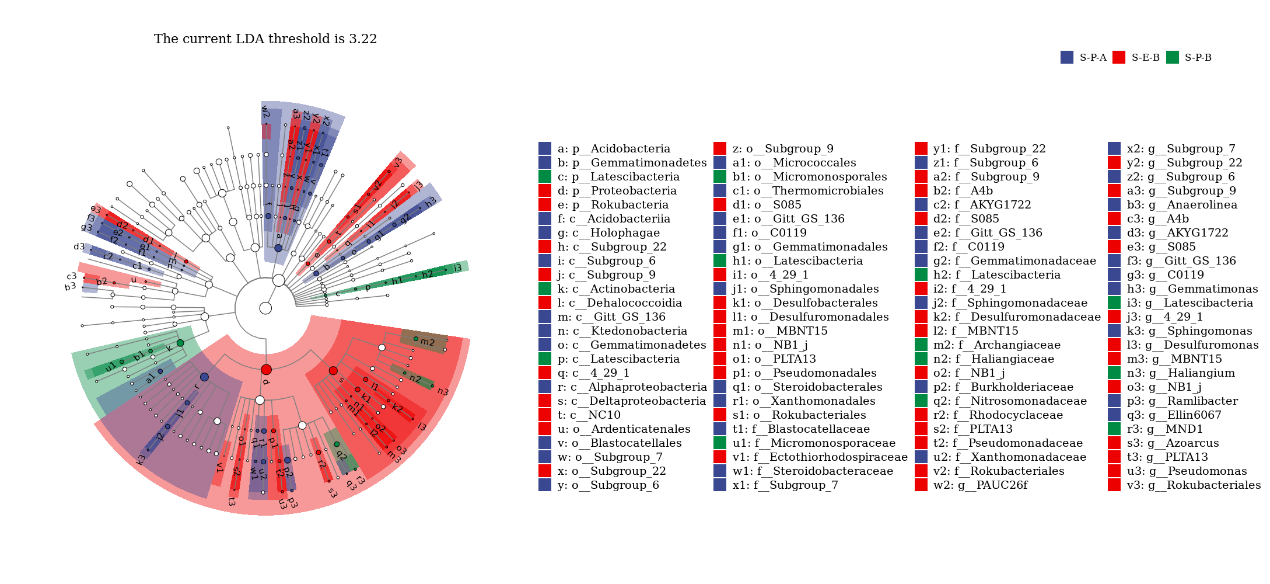


**Figure S2**. Lefse analysis for sediment bacteria community under different vegetations.

S-E-B: the subsurface sediment of *Erigeron canadensis* L; S-P-A: the surface sediment of *Phragmites australis (Cav.)* Trin. Ex Steud; S-P-B: the subsurface sediment of *Phragmites australis (Cav.)* Trin. Ex Steud.


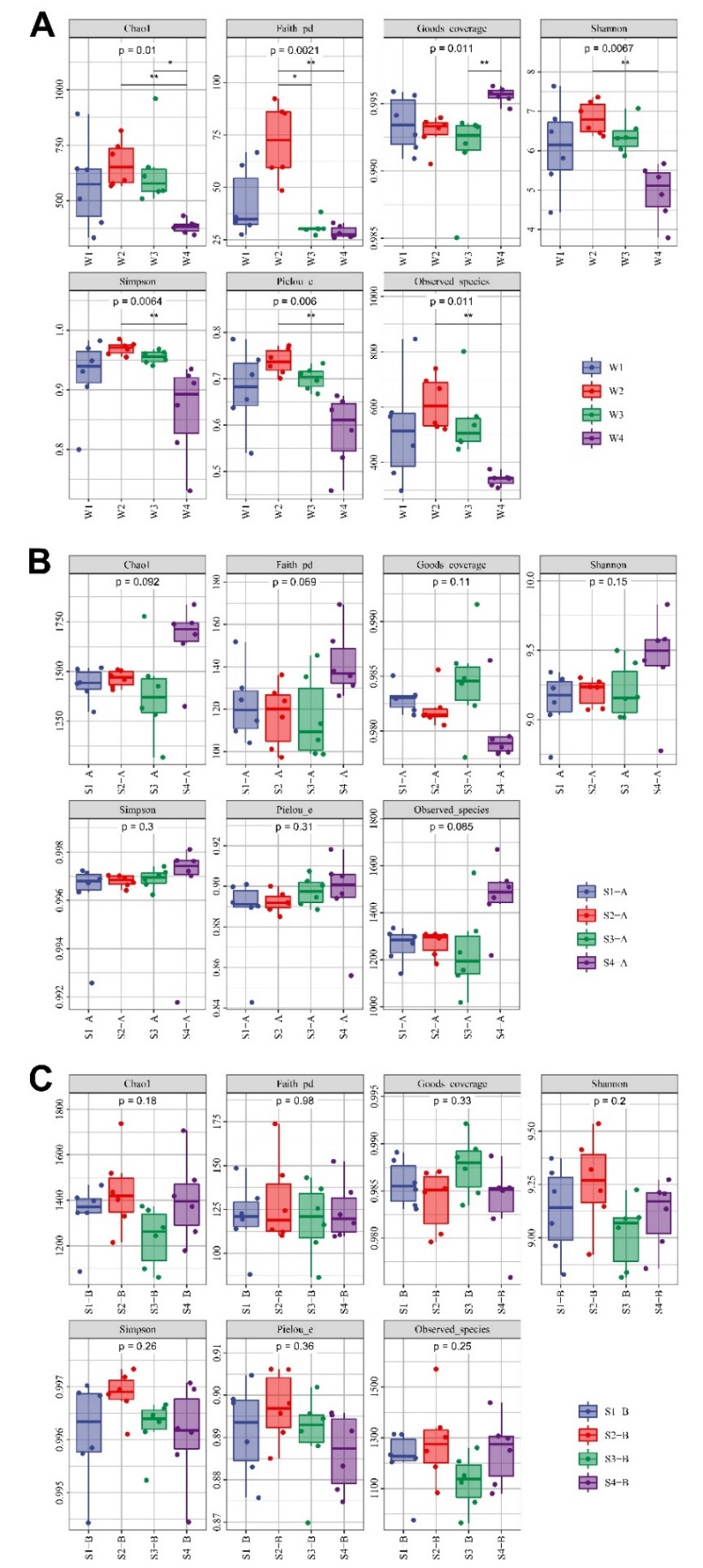


**Figure S3** The alpha diversity indices in the bacterial communities of water (A) and sediment (B and C) between the four times samples. W1: water sampled for the first time; W2: water sampled for the second time; W3: water sampled for the third time; W4: water sampled for the fourth time; S1: sediment sampled for the first time; S2: sediment sampled for the second time; S3: sediment sampled for the third time; S4: sediment sampled for the fourth time; A: surface sediment; B: subsurface sediment.


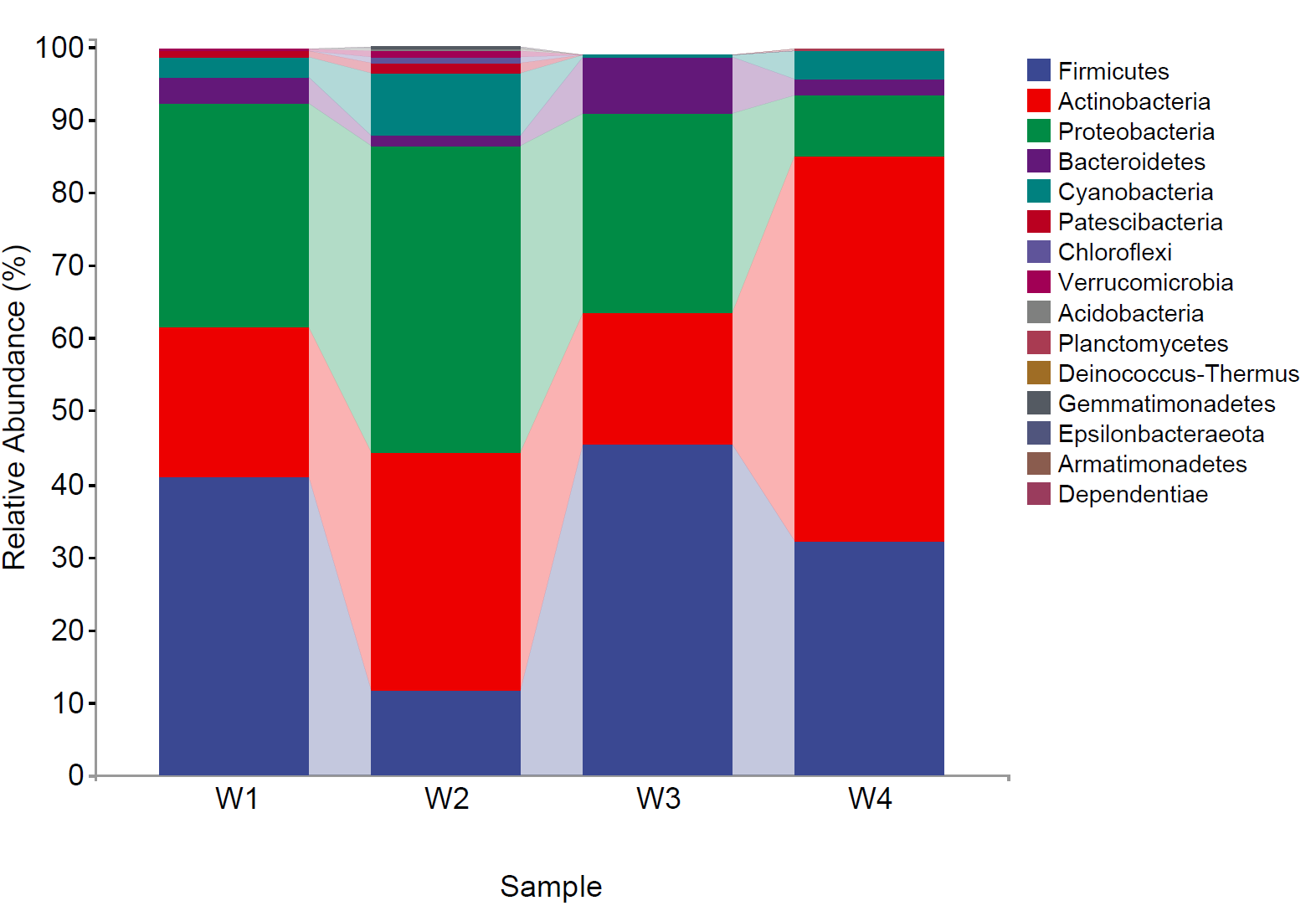


**Figure S4** The relative abundance of the dominant taxa in the water sampled for four times. W1: water sampled for the first time; W2: water sampled for the second time; W3: water sampled for the third time; W4: water sampled for the fourth time


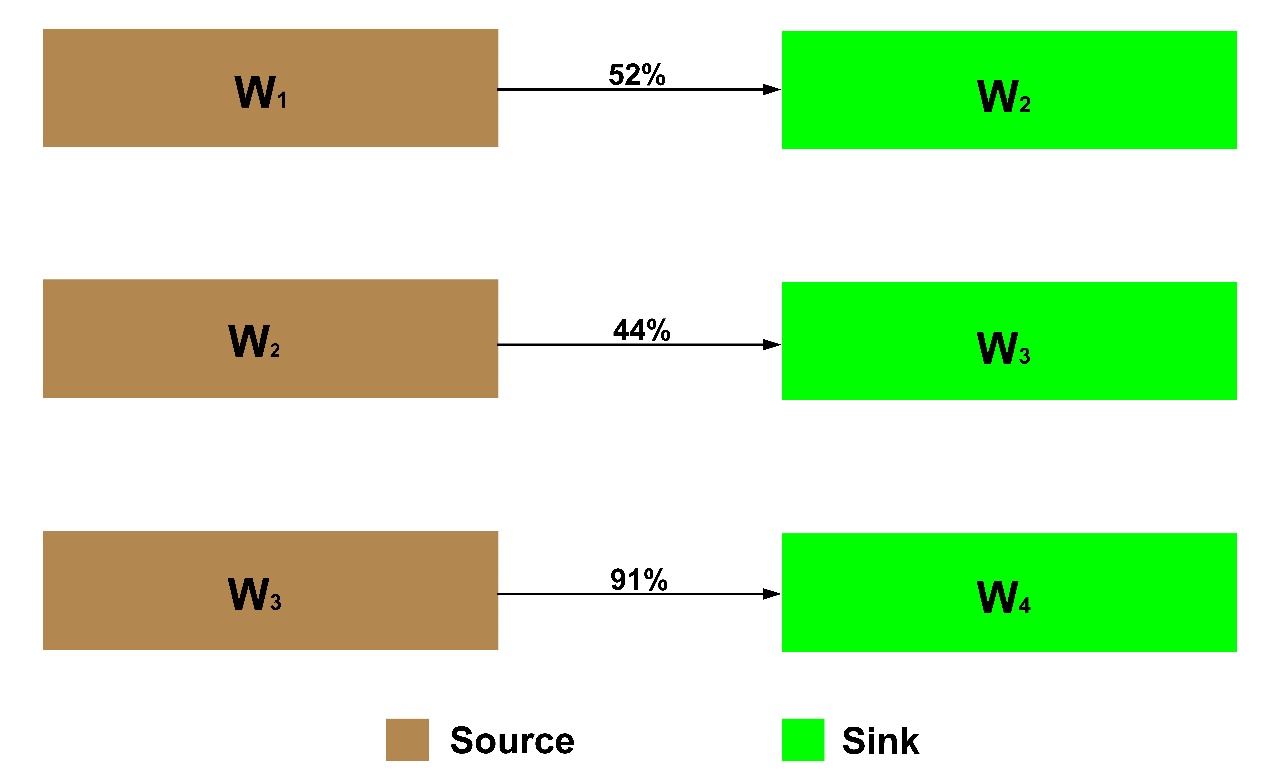


**Figure S5** The results of SourceTracker analysis show the contribution of bacteria ASV in the water collected four times. W1: water sampled for the first time; W2: water sampled for the second time; W3: water sampled for the third time; W4: water sampled for the fourth time.


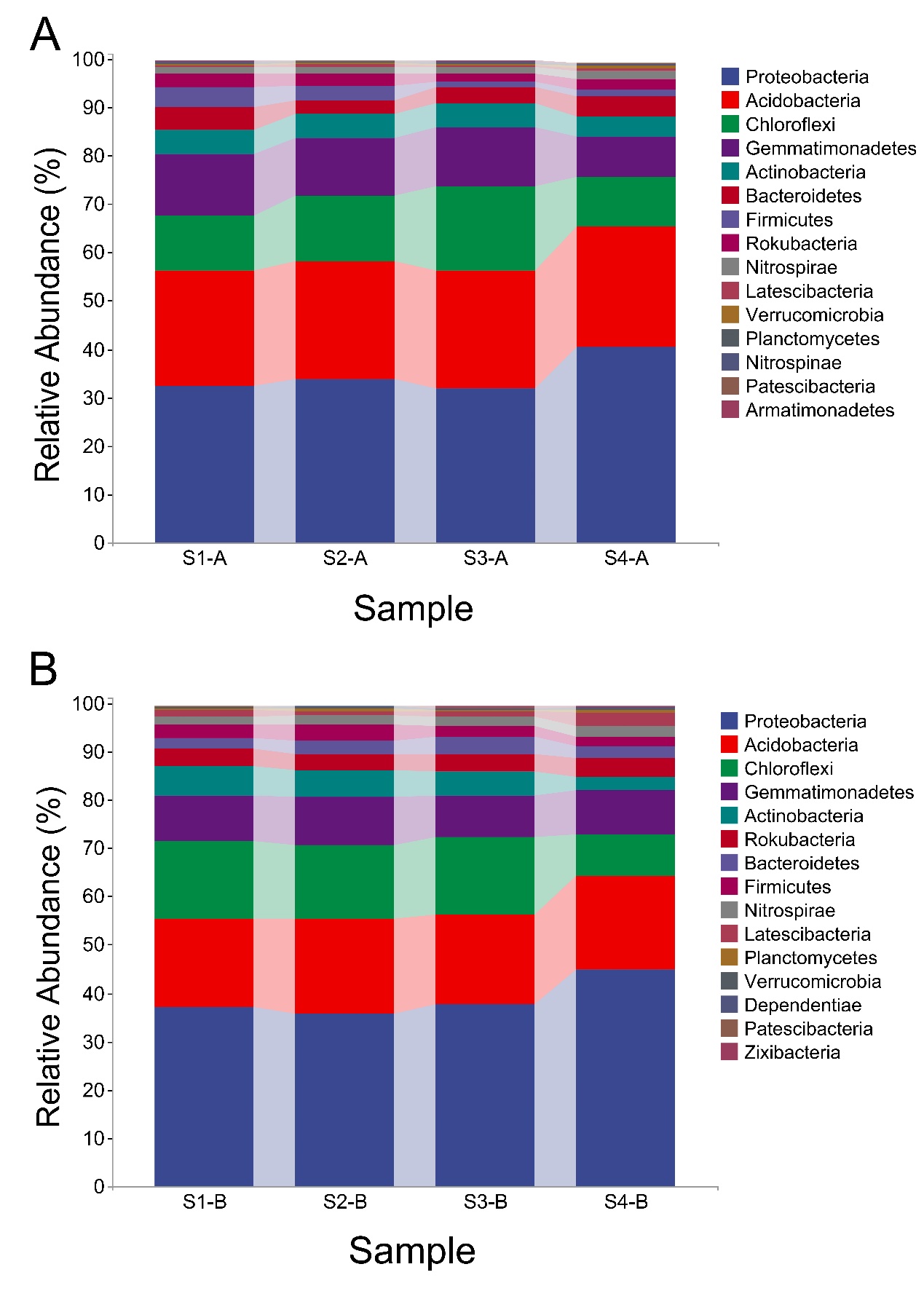


**Figure S6** The relative abundance of the dominant taxa in the in the surface and subsurface sediments sampled for four times. S1: sediment sampled for the first time; S2: sediment sampled for the second time; S3: sediment sampled for the third time; S4: sediment sampled for the fourth time; A: surface sediment; B: subsurface sediment.


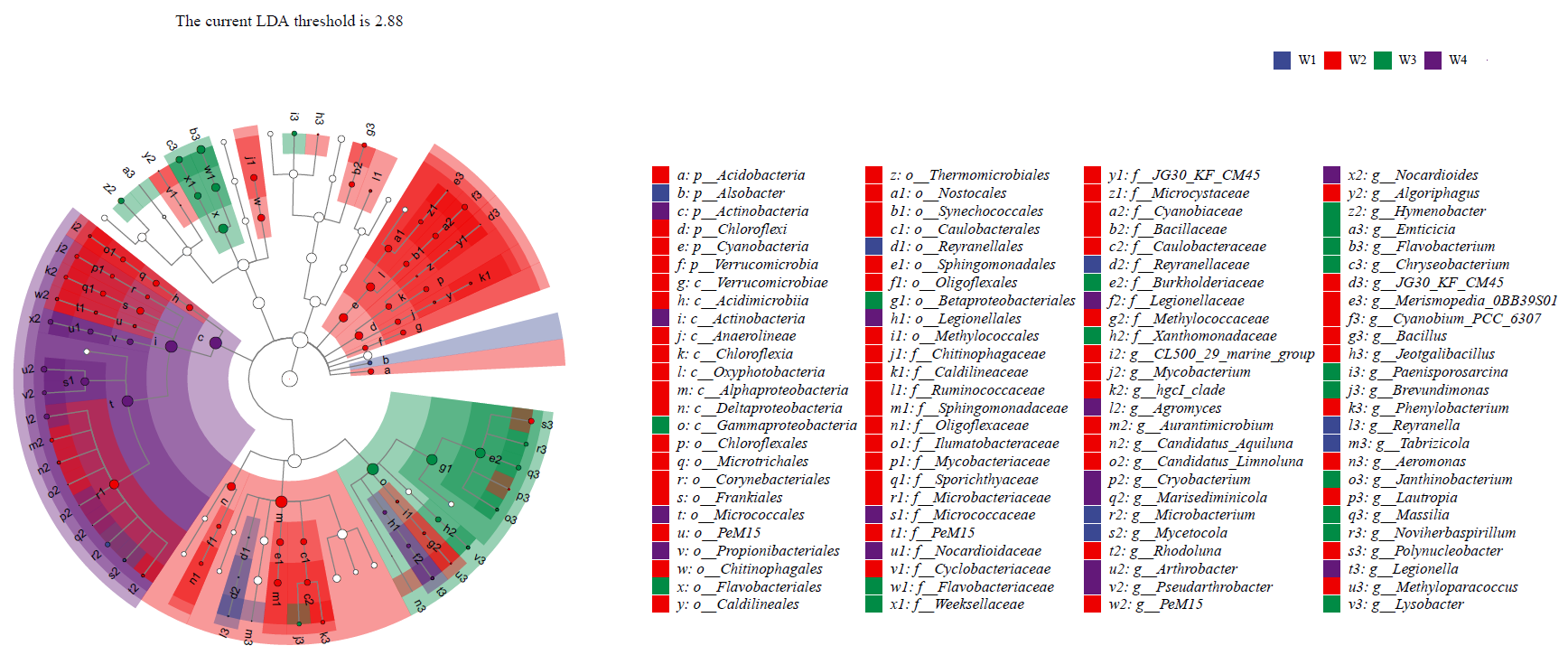


**Figure S7** Lefse analysis for bacteria community in the water sampled for four times.

W1: water sampled for the first time; W2: water sampled for the second time; W3: water sampled for the third time; W4: water sampled for the fourth time.

Table S1 Detailed arrangements of designated sink and source sites.

| **Sink sites** | **Source sites** |
| --- | --- |
| W (Water) | S-A (surface sediment) |
| W (Water) | S-B (subsurface sediment) |
| S-A (surface sediment) | W (Water) |
| S-B (subsurface sediment) | W (Water) |
| W2 (The water sampled for the second time) | W1 (The water sampled for the first time) |
| W3 (The water sampled for the third time) | W2 (The water sampled for the second time) |
| W4 (The water sampled for the fourth time) | W3 (The water sampled for the third time) |

**Table S2** Analysis of ANOSIM similarity between water and sediment.

| **Group1** | **Group2** | **R** | **pvalue** | **qvalue** |
| --- | --- | --- | --- | --- |
| All |  | 0.70 | 0.001 |  |
| Water | Surface sediment | 0.99 | 0.001 | 0.001 |
| Water | Subsurface sediment | 0.99 | 0.001 | 0.001 |
| Surface sediment | Subsurface sediment | 0.19 | 0.001 | 0.001 |

**Table S3** The relative contribution (%) of individual mechanism of soil microbial community assembly in water and sediment.

| **Bacteria** | Water | Surface sediment | Subsurface sediment |
| --- | --- | --- | --- |
| Variable selection (βΝΤΙ> +2) | 0 | 13.4 | 4.7 |
| Homogenous selection (βΝΤΙ< -2) | 69.9 | 30 | 40.2 |
| **Total selection (deterministic processes)** | **69.9** | **43.4** | **44.9** |
| Dispersal limitation (βΝΤΙ<\|2\| & RC> +0.95) | 4.7 | 5.4 | 20.7 |
| Dispersal homogenizing (βΝΤΙ<\|2\| & RC< -0.95) | 7.6 | 26.1 | 14.5 |
| Undominated (βΝΤΙ<\|2\| & RC< \|0.95\|) | 17.8 | 26.1 | 19.9 |
| **Total neutral (stochastic processes)** | **30.1** | **57.6** | **55.1** |

**Table S4** Topological properties of co-occurrence networks.

|  | **Water** | **Surface sediment** | **Subsurface sediment** |
| --- | --- | --- | --- |
| **Number of nodes** | 207 | 242 | 256 |
| **Number of edges** | 1678 | 374 | 1194 |
| **Edges/nodes** | 8.11 | 1.55 | 4.66 |
| **Avg number of neighbors** | 16.2 | 3.09 | 9.33 |
| **Network diameter** | 10 | 12 | 10 |
| **Characteristic path length** | 2.93 | 4.59 | 3.21 |
| **Clustering coefficient** | 0.56 | 0.29 | 0.41 |
| **Network density** | 0.08 | 0.01 | 0.04 |
| **Positive correlation** | 34.45％ | 35.29％ | 37.86％ |
| **Negative correlation** | 65.55％ | 64.71％ | 62.14％ |

**Table S5** Analysis of ANOSIM similarity between water and sediment of different plant communities. (W-E: the water of *Erigeron canadensis* L; W-P: the water of *Phragmites australis* (Cav.) Trin. Ex Steud; S-E-A: the surface sediment of *Erigeron canadensis* L; S-E-B: the subsurface sediment of *Erigeron canadensis* L; S-P-A: the surface sediment of *Phragmites australis* (Cav.) Trin. Ex Steud; S-P-B: the subsurface sediment of *Phragmites australis* (Cav.) Trin. Ex Steud.)

| **Group1** | **Group2** | **R** | **pvalue** | **qvalue** |
| --- | --- | --- | --- | --- |
| all | - | 0.07 | 0.08 | - |
| W-E | W-P | 0.07 | 0.08 | 0.08 |
| all | - | 0.24 | 0.001 | 0.004 |
| S-E-A | S-P-A | 0.17 | 0.003 | 0.002 |
| S-E-A | S-E-B | 0.28 | 0.001 | 0.013 |
| S-E-A | S-P-B | 0.10 | 0.013 | 0.002 |
| S-P-A | S-E-B | 0.51 | 0.001 | 0.002 |
| S-P-A | S-P-B | 0.25 | 0.001 | 0.012 |
| S-E-B | S-P-B | 0.21 | 0.01 | 0.004 |

**Table S6** Analysis of ANOSIM similarity between four sampling of water. (W1: water sampled for the first time; W2: water sampled for the second time; W3: water sampled for the third time; W4: water sampled for the fourth time.)

| **Group1** | **Group2** | **R** | **pvalue** | **qvalue** |
| --- | --- | --- | --- | --- |
| All |  | 0.60 | 0.001 |  |
| W1 | W2 | 0.61 | 0.003 | 0.003 |
| W1 | W3 | 0.45 | 0.004 | 0.004 |
| W1 | W4 | 0.37 | 0.001 | 0.003 |
| W2 | W3 | 1 | 0.003 | 0.003 |
| W2 | W4 | 0.99 | 0.002 | 0.004 |
| W3 | W4 | 0.54 | 0.003 | 0.004 |

**Table S7** Analysis of ANOSIM similarity between four sampling of soil. (S1: sediment sampled for the first time; S2: sediment sampled for the second time; S3: sediment sampled for the third time; S4: sediment sampled for the fourth time; A: surface sediment; B: subsurface sediment.)

| **Group1** | **Group2** | **R** | **pvalue** | **qvalue** |
| --- | --- | --- | --- | --- |
| All |  | 0.01 | 0.34 |  |
| S1-A | S2-A | 0.01 | 0.93 | 0.93 |
| S1-A | S3-A | -0.09 | 0.36 | 0.54 |
| S1-A | S4-A | 0.01 | 0.12 | 0.45 |
| S2-A | S3-A | 0.10 | 0.62 | 0.75 |
| S2-A | S4-A | -0.05 | 0.15 | 0.45 |
| S3-A | S4-A | 0.08 | 0.34 | 0.54 |
| All |  | -0.01 | 0.48 |  |
| S1-B | S2-B | -0.01 | 0.92 | 0.92 |
| S1-B | S3-B | -0.09 | 0.44 | 0.67 |
| S1-B | S4-B | -0.02 | 0.46 | 0.67 |
| S2-B | S3-B | -0.01 | 0.44 | 0.67 |
| S2-B | S4-B | -0.01 | 0.13 | 0.67 |
| S3-B | S4-B | 0.10 | 0.56 | 0.67 |

**Table S8** The relative contribution (%) of individual mechanism of bacterial community assembly of water sampled for four times. (W1: water sampled for the first time; W2: water sampled for the second time; W3: water sampled for the third time; W4: water sampled for the fourth time)

| **Bacteria** | W1 | W2 | W3 | W4 |
| --- | --- | --- | --- | --- |
| Variable selection (βΝΤΙ> +2) | 0 | 0 | 0 | 0 |
| Homogenous selection (βΝΤΙ< -2) | 80 | 73 | 100 | 100 |
| **Total selection (deterministic processes)** | **80** | **73** | **100** | **100** |
| Dispersal limitation (βΝΤΙ<\|2\| & RC> +0.95) | 0 | 0 | 0 | 0 |
| Dispersal homogenizing (βΝΤΙ<\|2\| & RC< -0.95) | 13.3 | 27 | 0 | 0 |
| Undominated (βΝΤΙ<\|2\| & RC< \|0.95\|) | 6.7 | 0 | 0 | 0 |
| **Total neutral (stochastic processes)** | **20** | **27** | **0** | **0** |
